# Impact of the BioFire FilmArray gastrointestinal panel on patient care and infection control

**DOI:** 10.1371/journal.pone.0228596

**Published:** 2020-02-06

**Authors:** Julian D. Machiels, Amelieke J. H. Cremers, Muriël C. G. T. van Bergen-Verkuyten, Sandra J. M. Paardekoper-Strijbosch, Kelly C. J. Frijns, Heiman F. L. Wertheim, Janette Rahamat-Langendoen, Willem J. G. Melchers

**Affiliations:** Department of Medical Microbiology & Radboudumc Center for Infectious Diseases, Radboud university medical center, Nijmegen, The Netherlands; Universita degli Studi di Parma, ITALY

## Abstract

**Objectives:**

Conventional routine PCR testing for gastrointestinal infections is generally based on pathogen related panels specifically requested by clinicians and can be erroneous and time consuming. The BioFire FilmArray gastrointestinal (GI) panel combines 22 pathogens into a single cartridge-based test on a random-access system, thereby reducing the turnaround time to less than 2 hours. We described the clinical impact of implementing the BioFire FilmArray on patients with gastroenteritis in our hospital.

**Methods:**

Patients attending a Dutch tertiary care center (Radboud University Medical Center), from whom stool samples were obtained, were eligible for inclusion. The clinicians selected one or a combination of different routinely performed PCR panels (bacterial panel, viral panel, clostridium testing, and three parasitic panels) based on clinical history and symptoms. All samples were in parallel tested with the FilmArray. We retrospectively collected patient data regarding infection control and patient management to assess the potential impact of implementing the FilmArray.

**Results:**

In total 182 patients were included. Routine PCR detected one or more pathogens in 52 (28.6%) patients compared to 72 (39.6%) using the FilmArray. Turnaround time (including transport) decreased from median 53 hours for the routine PCR to 16 hours for the FilmArray. Twenty-six patients could have been removed from isolation 29 hours sooner, 3.6 antibiotic days could have been saved and in five patients additional imaging testing (including colonoscopies) could have been prevented.

**Conclusion:**

The theoretical implementation of the BioFire FilmArray GI panel in patients with clinical suspicion of gastroenteritis resulted in a significant better patient management.

## Background

Diarrhea is a common cause of morbidity and hospital admission. In the Netherlands, the annual incidence of gastrointestinal (GI) infection is estimated to be 4.5 million episodes with an estimated cost of 611–695 million euro.[1, 2] Admitted patients with symptoms of gastroenteritis (diarrhea, vomiting) are isolated until the result of the diagnostic test is known or another, non-infectious, cause has been identified. Also, empirically started therapy can be adjusted or specific treatment can be started based on symptoms and test results.[3]

The diagnosis of infectious gastroenteritis is generally driven by pathogen directed molecular panels. These panels are pathogen specific and based on expected bacterial, viral or parasitic causes. Which panel to request (viral, bacterial or parasitic) is determined by the history of the patient, symptoms, and the clinical assessment of the physician. The pattern of clinical symptoms however cannot differentiate with sufficient accuracy between the various causes of gastroenteritis.[4–6] Thus, the current diagnostic approach may lead to misdiagnosed cases, which potentially affects clinical care.[7] Furthermore, depending on the laboratory routine, turn-around times of one or more days is common practice, during which a patient is isolated with empirical therapy or no therapy at all.[8]

The BioFire FilmArray GI Panel (BioFire Diagnostics) contains 22 of the most common GI pathogens in a single panel. With this random access test, the hands-on time is reduced to around 3 minutes and the test has a turnaround time (TAT) of about one hour.[9]

In this study we evaluated the potential impact of rapid microbial diagnosis in gastrointestinal disease on infection control and patient care.

## Methods

### Ethics approval

The study was approved by the Medical Ethics Committee of the Radboud University Medical Center. No consent was obtained (data was analyzed anonymously).

### Population

The study was conducted from December 2017 till July 2018 at the Radboud University Medical Centre, a tertiary care, academic medical center in the Netherlands. Stool samples submitted for analyses of GI infection with routine PCR were parallel tested on the BioFire FilmArray. Data regarding infection control and patient management of included patients were recorded retrospectively. Only patients who were seen at our medical center and of whom fecal samples for routine diagnostics had been sent in, were eligible for inclusion. Patients where only testing for *C*. *difficile* toxin (CDT) was requested were not included, but patients where CDT was requested in combination with a different panel, were included.

No specific criteria for requesting a certain panel on the routine PCR were used. The physicians selected one or a combination of panels based on his or her assessment of the clinical symptoms together with the history of the patient. The available panels are: a (I) bacterial panel, with CDT on demand in addition, a (II) basic parasitic panel, a (III) parasitic panel for travelers, a (IV) parasitic panel for immunocompromised patients and a (V) viral panel (Table 1).

**Table 1:** Pathogens included in the BioFire FilmArray GI Panel and the routine PCR.

	Routine PCR	FilmArray GI Panel
**Bacterial panel**		
*Salmonella*	X	X
*Campylobacter jejuni*	X	X
*Campylobacter coli*	X	X
*Campylobacter upsaliensis*		X
*Yersinia enterocolitica*	X	X
CDT		X
*Plesiomonas shigelloides*		X
*Vibrio spp*		X
*Vibrio cholera*		X
EAEC		X
EPEC		X
ETEC		X
STEC	X	X
*E*. *coli* O157		X
EIEC/Shigella spp	X	X
**Viral panel**		
*Norovirus*	X	X
*Enterovirus*	X	
*Parechovirus*	X	
*Adenovirus*	X	
*Adeno* serotype 40/41	X	X
*Bocavirus*	X	
*Rotavirus*	X	X
*Astrovirus*	X	X
*Sapovirus*	X	X
**Basic parasitic panel**		
*Dientamoeba fragilis*	X	
*Giardia lamblia*	X	X
*Cryptosporidium spp*	X	X
**Travelers parasitic panel**		
*Entamoeba histolytica*	X	X
*Cyclospora cayetanensis*	X	X
*Strongyloides stercoralis*	X	
**Opportunistic parasitic panel**		
*Cystoisospora belli*	X	
*Encephalitozoon spp*	X	
*Enterocytozoon bieneusi*	X	

List of pathogens included in the routine PCR and the BioFire FilmArray.

X: included in panel.

CDT: *Clostridium difficile* toxin; STX: Shiga-toxin; EAE: Attaching-And-Effacing

EAEC: *Enteroaggregative E*. *coli*; EPEC: *Enteropathogenic E*. *coli*; ETEC: *Enterotoxigenic E*. *coli*; STEC: *Shiga-like toxin-producing E*. *coli*; EIEC: *Shigella/Enteroinvasive E*. *coli*.

### Test method

In case a parasitic panel was requested, a small quantity of the feces was transferred into a bead bottle containing beads and 1 mL phosphate-buffered saline (PBS). The sample was beated twice for 20 seconds. In case a bacterial or viral panel was requested, a small quantity was transferred into a tube containing 2,5 mL PBS. This was vortexed for 1 minute, let to rest for 10 minutes at room temperature and then vortexed again for another 1 minute. This was then centrifuged at 3000 rpm for 2 minutes. The suspension was pipetted into another tube. Samples for CDT were tested without any processing beforehand. All samples were either tested the same day, or frozen at -80°C until the test was run. A 195 μL sample for the routine PCR was used. All routine PCR’s were run on the Roche Aurora Flow in a monoplex or multiplex PCR assay with primers and probes as previously described.[10–18] The bacterial and parasitic panels were batch wise run once daily five days per week, the viral panel was run thrice weekly (Monday, Wednesday and Friday). When a test run was inhibited twice, the test was reported “not interpretable” and the clinician was asked to send in a new stool sample.

CDT testing was primarily done using the Techlab C.DIFF QUIK CHEK COMPLETE^®^ (Alere). Only in the case when *Clostridium* antigen was positive and the toxin test was negative, or vice versa, the sample was tested with the Cepheid GeneXpert^®^ XVI Xpert *C*. *difficile* for confirmation.

The BioFire FilmArray GI Panel detects thirteen bacteria, five viruses and four parasites (Table 1).[9] The assay was performed according to the manufacturer’s instructions. Test results of the FilmArray were not reported to the physician during the study.

Data collected from electronic patient charts included age, sex, comorbidity, length of hospital admission, use of antibiotics, additional diagnostics performed related to GI symptoms, isolation days, time of test ordering, arrival at the laboratory, initiation of the test, completion of the test and time of reporting to the physician. We used the time of test ordering as a proxy for the time the feces were collected because the exact moment of collection was unknown. Nosocomial diarrhea was defined as the onset of gastrointestinal symptoms 48 hours after admission to the hospital. We used a TAT of 1.5 hours for the FilmArray for calculations of the total TAT’s. Action taken by the clinician (e.g. patient removed from isolation) within 24 hours after a test result was defined as an action related to the test result.

### Statistics

For the statistical analyses we used IBM SPSS Statistics 23. All data are reported as mean ± 1 standard deviation (SD) or as median and range. Missing data were treated as missing. Statistical significance was assessed using the Wilcoxon test, the McNemar’s test, Fisher’s exact test or the Chi Square test where appropriate. A probability (p) value of ≤0.05 was considered statistically significant.

## Results

### Baseline characteristics

A total of 182 patients were included. All stool samples were tested simultaneously by routine PCR and on the FilmArray GI panel. Median age of the patients was 45 (0–97) years. 120 (65.9%) patients were hospitalized for a median of 8.5 days (1–206). Of the hospitalized patients, 98 (82.4%) were put in isolation with a median of 4 (1–57) days in isolation per patient (Table 2).

**Table 2:** Baseline characteristics.

Patients, n	182
Male, %	47.8
Median age, years (range)	45 (0–97)
Patients hospitalized, n (%)	120 (65,9)
Number of days hospitalized per patient, median (range)	8.5 (1–206)
Isolated, n (%)	98 (82,4)
Number of days isolated per patient, median (range)	4 (1–57)
Nosocomial, %	30.5
Comorbidity, n (%)	140 (76.9)
IBD, n (%)	21 (11.5)
IBS, n (%)	3 (1.6)
Solid malignancy,n (%)	23 (12.6)
Hematology (including malignancies and SCT), n (%)	30 (16.5)
Organ transplant, n (%)	14 (7.7)
Other, n (%)	56 (30.8)
Antibiotics prescribed, n (%)	40 (22.6)
Number of days on antibiotics, median (range)	8 (1–38)
All cause mortality, n (%)	18 (9.9)

Data are reported as mean ± 1 SD or as median with range.

IBD: irritable bowel syndrome; IBS: inflammatory bowel syndrome.

**Fig 1:**
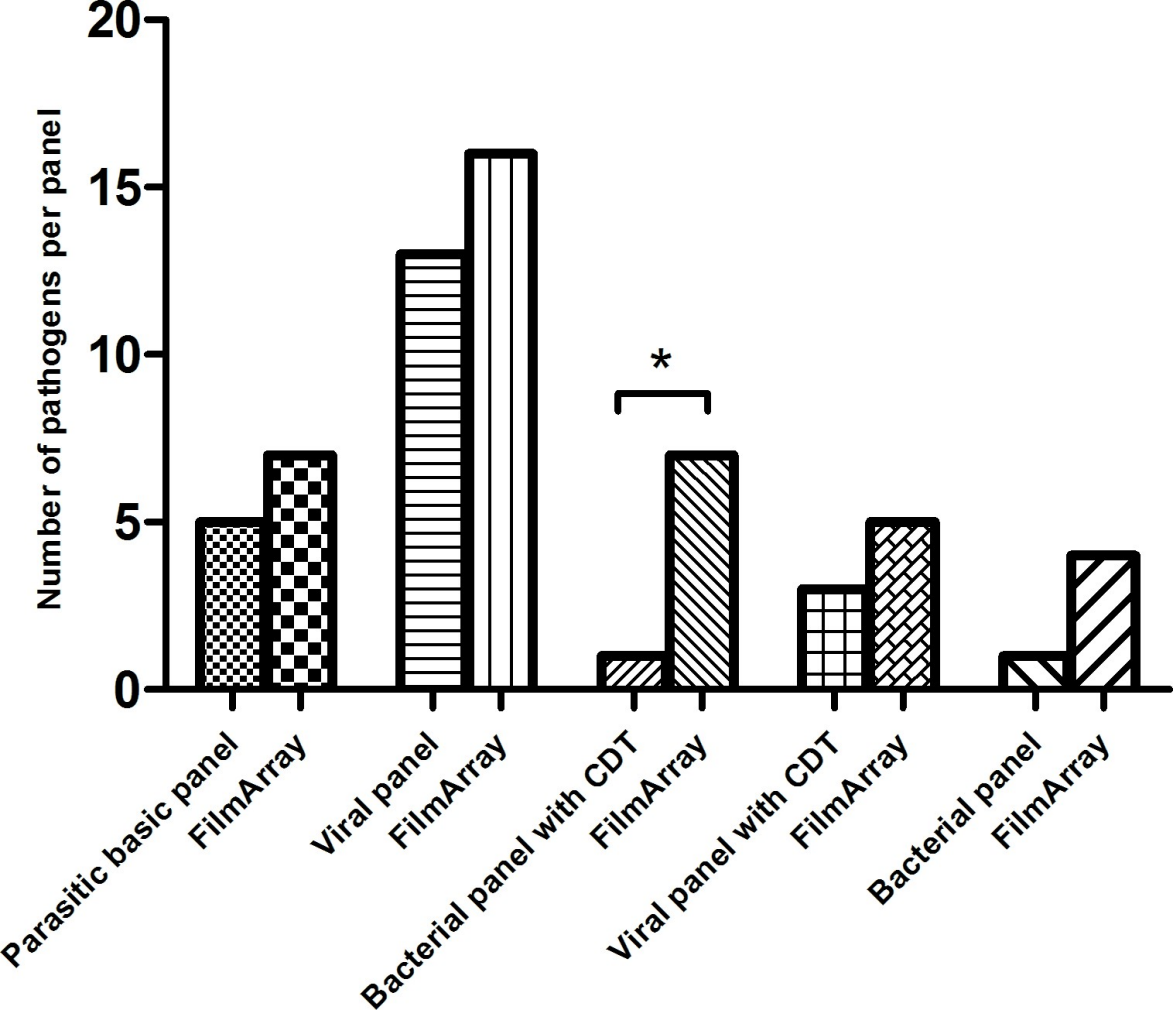
Additional pathogens found by the FilmArray compared to the mostly requested panel or combination of panels on the routine PCR. Number of pathogens found in the FilmArray and the mostly requested panels on the routine PCR. * significant result.

The most requested (combination) of panels were the basic parasitic panel (n = 24), viral panel (n = 42), bacterial panel combined with CDT (n = 17) and viral panel combined with CDT (n = 17) (S1 Fig). In 56% of all the patients more than one panel was requested by the clinician. Fig 1 shows the additional pathogens detected by the FilmArray when compared to the most frequently requested combination of panels. In each instance the FilmArray detected more pathogens. No inhibition was found using the FilmArray.

### Concordance testing

Routine PCR detected at least one pathogen in 52 (28.6%) patients, compared to 72 (39.6%) pathogens detected by the FilmArray (p = 0.001). Routine PCR detected in 48/52 (92.3%) patients one pathogen and in 4/52 (7.7%) patients two pathogens. The FilmArray detected in 50/72 (69.4%) patients one pathogen, in 19/72 (26.4%) patients two pathogens and in 3/72 (4.2%) patients three or more pathogens. FilmArray detected 29 pathogens that were either negative, not tested or “not detected because of inhibition” with the routine PCR. This included eight CDT, seven *Noroviruses*, ten *EPEC* and three *Campylobacters*.

The FilmArray detected 26 pathogens (mainly CDT and *Norovirus*) there were on a panel included in the routine PCR, but not ordered by the clinician (Table 3).

**Table 3:** Pathogens in the FilmArray compared to non-ordered panels on the routine PCR.

	Pathogen	Number of positive pathogens
Bacterial pathogens	Campylobacter	3
	Yersinia	1
	STEC	1
	CDT	9
Viral pathogens	Noro	5
	Adeno 40/41	1
	Rota	1
	Astro	1
	Sapo	3
Parasitic pathogens	Cyclospora	1

Positive pathogens found in the FilmArray compared to pathogens that are included in the routine PCR but were not ordered.

CDT: *Clostridium difficile* toxin; STEC: *Shiga-like toxin-producing E*. *coli*

The FilmArray detected 19 pathogens that were not included in any of the routine PCR panels. Positive pathogens included 12 EPEC and six EAEC (Table 4).

**Table 4:** Pathogens in the FilmArray not included in the routine PCR.

	Pathogen	Number of positive pathogens
Bacterial pathogens	Plesiomonas	1
	EAEC	6
	EPEC	12

Pathogens and number of positive pathogens in the routine PCR that are not included in the FilmArray.

EAEC: *Enteroaggregative E*. *coli*; EPEC: *Enteropathogenic E*. *coli*; ETEC: *Enterotoxigenic E*. *coli*

The routine PCR detected 12 pathogens (mainly D. fragilis) that were not included in the FilmArray (Table 5).

**Table 5:** Pathogens in the routine PCR that are not included in the FilmArray.

	Pathogen	Number of positive pathogens
Viral pathogen	Bocavirus	1
	Enterovirus/parecho	1
	Adenovirus	2
Parasitic pathogen	D. fragilis	8

Pathogens in the routine PCR that are not included in the FilmArray and the number of positive results.

**Fig 2:**
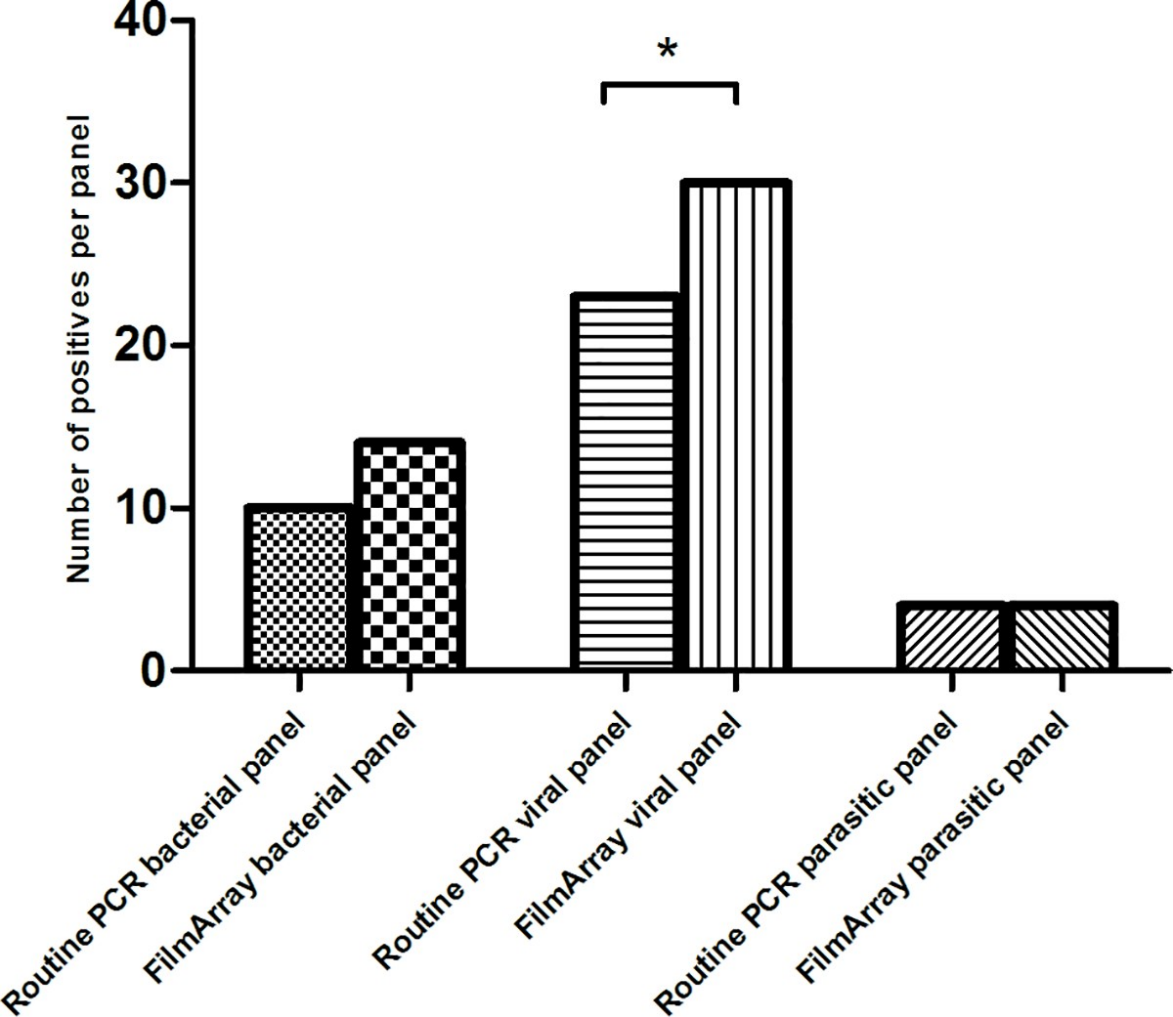
Additional pathogens found by the FilmArray compared to the panel on the routine PCR. Both the panel on the routine are matched and contain the same pathogens. Number of additional pathogens found that are included in the FilmArray and the routine PCR. * significant result.

The FilmArray detected nine pathogens that were on an ordered panel in the routine PCR but not detected by the routine PCR. This included six pathogens were the routine PCR was negative and three pathogens were the result in the routine PCR was “not interpretable” because of inhibition. The most common pathogens not detected or inhibited in the routine PCR, but positive in the FilmArray was *Norovirus* (n = 4, three times not interpretable in routine PCR). On the other hand, only one pathogen (CDT) was positive in the routine PCR and negative in the FilmArray. (Fig 2 and S2 Fig).

### Turnaround times

**Fig 3:**
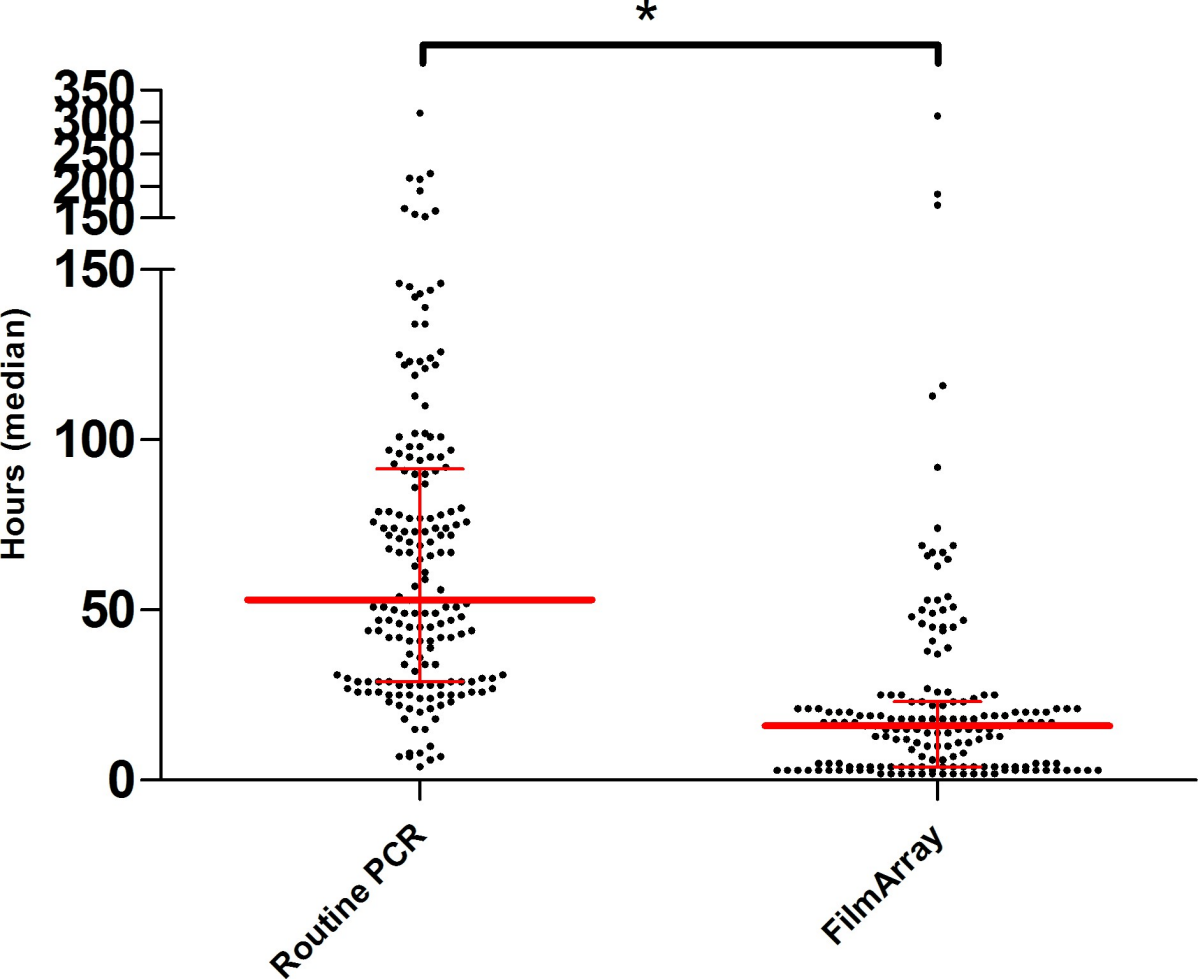
Turnaround times from collection to reporting in median hours. Turnaround times from collection to reporting to the clinician in median hours. Time of ordering was used as a proxy for time of collection.

Fig 3 show the turnaround times. Time from collection to reporting was 53 hours (median) using routine PCR compared to 16 hours (median) for the FilmArray. Runtime for the routine PCR was 4 hours (median). Using the FilmArray testing was 29 hours (median) faster compared to routine PCR (Fig 3).

### Clinical impact

**Fig 4:**
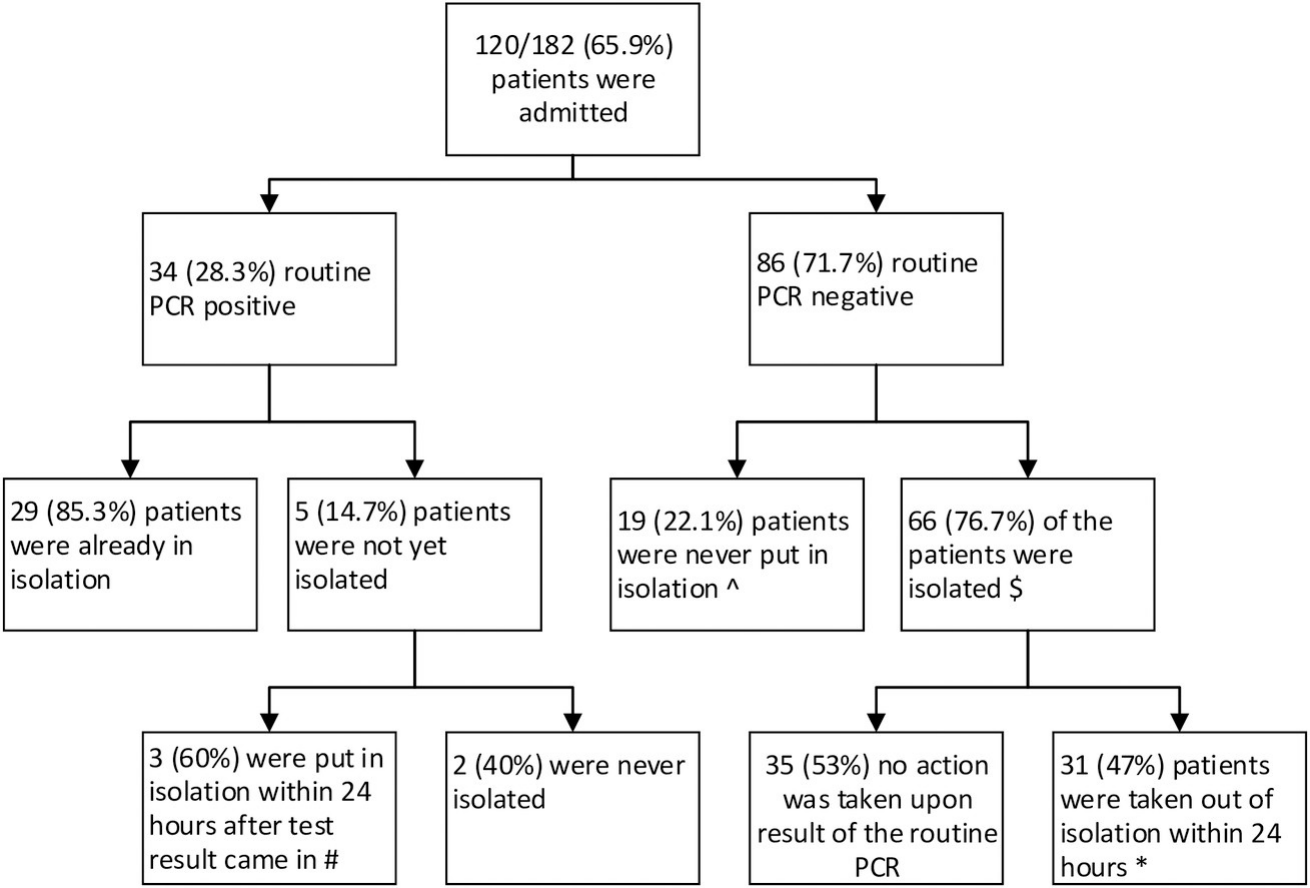
Flow chart of the patients admitted to the hospital and their isolation status according to the rest result of the routine PCR. Flow chart of the patients admitted to the hospital and their isolation status according to the rest result of the routine PCR. $ in 1 patient isolation status was missing. # 3 were positive in the FilmArray. * 5 patients were tested positive in the FilmArray and 26 patients tested negative. ^ 4 patients were tested positive in FilmArray.

120 of the 182 patients (65.9%) were admitted to the hospital. The ordered routine PCR was negative in 86/120 patients (71.7%). Of the 86 admitted patients with a negative PCR, 66 (75.6%) were isolated. 31 of the patients put in isolation but with a negative PCR, were removed from isolation within 24 hours after the test result was reported to the clinician (Fig 4). In 26 of these 31 patients (83.9%) the FilmArray was also negative. In five patients the FilmArray found one or more pathogens (two *Norovirus*, one *Campylobacter*, one EAEC, one EPEC and one *Cyclosporidium*) and therefore should have been kept in isolation.

In 34 (28.3%) of the admitted patients the ordered routine PCR was positive. Of these patients, five were not yet isolated when the test result was reported to the clinician. Three patients were put in isolation within 24 hours after the test result became available and all three patients were also positive in the FilmArray (two rotavirus, two *Campylobacters*, one *CDT*). In two of the 34 admitted patients with a positive routine PCR the FilmArray tested negative, however the pathogens detected in the routine PCR were not included in the FilmArray (*Entero*-/*Parechovirus* and *Adenovirus* non-40/41). In addition, five patients with a negative routine PCR but with a positive FilmArray had undergone additional diagnostic procedures (two ultrasounds of the abdomen, one gastroscopy and three colonoscopies). The FilmArray detected one *Sapovirus*, one *Campylobacter*, one *Plesiomonas*, one *C*. *difficile* and three *EPEC* in these five patients.

If the FilmArray would have been implemented for routine molecular testing, additional diagnostic procedures and isolation days could have been prevented. In this study 26 patients were removed from isolation when the routine PCR result was reported negative to the clinician. If the FilmArray would have been used as a first line diagnostic system, for each of these patients a median of 29 hours would have been gained, thus saving 31.4 (range 1–187) days in isolation. Antibiotic therapy could have been stopped in three patients, this would have saved 3.6 (range 0.1–21.6) days on antibiotics. Five patients had undergone additional diagnostic procedures that could have been prevented. Five patients were wrongly removed from isolation due to a negative routine PCR.

In the outpatient population, we found no impact on either antibiotic therapy or the number of prevented diagnostic procedures (data not shown).

## Discussion

In this study we evaluated the potential impact of rapid microbial diagnosis in gastrointestinal disease on infection control and patient care. In line with previously conducted studies, the diagnostic yield was higher using the FilmArray GI panel: one or more pathogen was detected in 39.6% of the patients compared to 28.6% by routine PCR.[8, 19, 20] Furthermore, The FilmArray detected pathogens that were included in the routine PCR panels, but were not ordered by the clinician and it detected pathogens that were not on any of the routine PCR panels. Using the FilmArray, time to result was significantly shortened compared to routine PCR. Consequently, more than 31 days in unnecessary isolated care could have been prevented. Also, implementing the FilmArray could have prevented additional invasive diagnostics procedures in five of the 131 patients.

Five patients were removed from isolation because of a negative routine PCR but with a positive FilmArray result. Based on the detected pathogens, given our hospital policy, at least two of five would need isolated care, and were thus wrongfully removed from isolation.

To our knowledge, there has only been one previously conducted study on the impact of the FilmArray on isolation days in GI patients.[21] In this study 24.5% of the patients with a negative FilmArray could have been removed from isolation due to a negative panel on the FilmArray. 60% of the patients who had unexpected pathogen was not isolated at the time of testing. In our study, 83.8% of the isolated patients with a negative routine PCR could have been removed earlier from isolation had the FilmArray been implemented.

The advantages of the commercial assay in analytical sensitivity and turnaround time over conventional culture-based diagnostics have been described by Cybulski et al.[20] In our study we also found that a faster TAT leads to better patient management and an improved analytical sensitivity compared to our routine PCR. Unfortunately, the design of our study did not allow to analyze the impact in targeted antibiotic treatment because we were not able to differentiate empirical antibiotic use versus targeted antibiotic use.

Clinicians in our hospital request specific panels based on history taking, symptoms and clinical assessment. With our data we were able to objectively judge this strategy since the clinician was not aware that the FilmArray GI panel was performed. The FilmArray detected pathogens that could have been detected in the routine PCR but were not requested by the clinician. Also, the FilmArray detected pathogens that were not included in the routine PCR. This means that in many instances the correct pathogens were missed because the routine PCR was used. Based on these data we conclude that the clinical symptoms are not an accurate predictor to differentiate bacterial, viral and parasitic causes of GI. The current diagnostic approach, where a clinician requests certain panels, may lead to false-negative or misdiagnosed cases, as is previously reported.[6]

Testing for CDT is controversial, international guidelines differ somewhat in their recommendations. The European guideline recommends testing for CDT in a two-step algorithm (nucleic acid test or GDH Enzyme Immuno Assay (EIA) followed by Toxin A/B EIA).[22] The IDSA guideline recommends that a clinician may use only a nucleic acid test when there are pre-agreed clinical criteria.[23] Nucleic acid tests are more sensitive and less specific for C. difficile infection, while toxin immunoassays are less sensitive but somewhat more specific.[22] However, either type of assay may be positive in patients with asymptomatic carriage, as well as in patients with symptomatic infection.[24] Nucleic acid testing detects more cases of symptomatic infection than an immunoassay, because it is more sensitive, and patients being evaluated for gastroenteritis in the hospital setting are presumably symptomatic. Therefore, it is likely that at least some hospitalized patients with C. difficile detected by FilmArray have true C. difficile infection. However, patients with infectious gastroenteritis caused by a different pathogen may also be asymptomatic C. difficile carriers. We therefore recommend that when the FilmArray detects C. difficile, follow-on testing by Toxin A/B EIA should be performed.

There has been some debate about the additional value of large multiplex testing in GI disease.[25, 26] In a recent Health Technology assessment conducted by Freeman et al. in the United Kingdom, the FilmArray was not cost-effective compared to routine PCR testing.[26] However, the routine PCR was based on an algorithm with consecutive testing of pathogens, whereas in our hospital the clinician is asked to select (a combination of) panels based on history taking and symptoms.[27]

Although the FilmArray GI panel is wide, it does not cover all gastrointestinal parasites. A number of pathogens that are included in the routine PCR are not included in the FilmArray GI panel. A few of these pathogens are included in our routine PCR to detect carriership or are used as proxy for invasive disease (e.g. *entero*-/*parechovirus*) and do not necessarily cause gastroenteritis. However, Bocavirus, D. fragilis and a few other parasites are not included in the FilmArray and these could be clinically relevant pathogens, although the pathogenicity of *Dientamoeba fragilis* has not been fully elucidated.[28] So, in special cases, a negative FilmArray result may therefore require additional feces testing (e. g. microscopy). The FilmArray showed advantages over using the routine PCR or conventional culture in both sensitivity and turnaround time.[20] One downside of using only culture-independent diagnostic testing such as the FilmArray that it does not allow for susceptibility testing for epidemiological investigations.[29] In our hospital we therefore culture feces with positive routine PCR for a bacterial pathogen.

The main strength of this study is that we tested all samples in the routine PCR and on the FilmArray, with a chart review for the clinical characteristics of the patients. We then analyzed the potential impact that implementing the FilmArray could have had on patient care. Limitations of this study include the small patient population and the retrospective, single center design. Also, as a tertiary care hospital, where most patients had severe comorbidities which might have led to an underestimation of the impact because many patients were isolated or underwent additional diagnostics due to their underlying comorbidity. Furthermore, because of the retrospective design of this study, we were not able to analyze targeted antibiotic use. Our study focused on the direct impact on patient care based on the PCR panels that were requested by the clinicians and not on validation the BioFire. Therefore, we did not perform discrepancy analyses on disconcordant results. Discrepancy studies have been performed previously.[30]

In conclusion, the BioFire FilmArray GI panel is a broad, multiplex gastrointestinal panel with a rapid turnaround time, resulting in a significant reduction of unnecessary isolation days, antibiotic therapy and prevention of additional diagnostic procedures in patients with clinical suspicion of infectious gastroenteritis.

## Supporting information

S1 Fig Panels or combination of panels requested on the routine PCR. Overview of the requested panels by the clinician, the combinations and the percentages.
(TIF)

S2 Fig Concordance per pathogen between the FilmArray and the routine PCR. Overview of the concordance between the routine PCR and the FilmArray for the pathogens that are included in both PCR systems.
(TIF)

S1 File (PDF)

## References

[1] de Wit M.A., Koopmans M.P., Kortbeek L.M., Wannet W.J., Vinje J., van Leusden F., et al, Sensor, a population-based cohort study on gastroenteritis in the Netherlands: incidence and etiology. Am J Epidemiol, 2001 154(7): p. 666–74. 10.1093/aje/154.7.666 11581101

[2] Friesema I.H., Lugner A.K., and van Duynhoven Y.T., Costs of gastroenteritis in the Netherlands, with special attention for severe cases. Eur J Clin Microbiol Infect Dis, 2012 31(8): p. 1895–900. 10.1007/s10096-011-1518-1 22228374

[3] Bos J., Schultsz C., Van Gool T., Bauer M., and Prins J., Swab guideline antimicrobial therapy in acute infectious diarrhea. 2014.

[4] Mattila L., Clinical features and duration of traveler's diarrhea in relation to its etiology. Clin Infect Dis, 1994 19(4): p. 728–34. 10.1093/clinids/19.4.728 7803639 PMC7109962

[5] Riddle M.S., DuPont H.L., and Connor B.A., ACG Clinical Guideline: Diagnosis, Treatment, and Prevention of Acute Diarrheal Infections in Adults. Am J Gastroenterol, 2016 111(5): p. 602–22. 10.1038/ajg.2016.126 27068718

[6] Stockmann C., Rogatcheva M., Harrel B., Vaughn M., Crisp R., Poritz M., et al, How well does physician selection of microbiologic tests identify Clostridium difficile and other pathogens in paediatric diarrhoea? Insights using multiplex PCR-based detection. Clin Microbiol Infect, 2015 21(2): p. 179.e9–15. 10.1016/j.cmi.2014.07.011 PMC4330102 25599941

[7] Beersma M.F., Sukhrie F.H., Bogerman J., Verhoef L., Mde Melo M., Vonk A.G., et al, Unrecognized norovirus infections in health care institutions and their clinical impact. J Clin Microbiol, 2012 50(9): p. 3040–5. 10.1128/JCM.00908-12 22785184 PMC3421828

[8] Beal S.G., Tremblay E.E., Toffel S., Velez L., and Rand K.H., A Gastrointestinal PCR Panel Improves Clinical Management and Lowers Health Care Costs. J Clin Microbiol, 2018 56(1). 10.1128/JCM.01457-17 PMC5744222 29093106

[9] *FilmArray^®^ Panels, Gastrointestinal Panel* 26-07-2018]; Available from: https://www.biofiredx.com/products/the-filmarray-panels/#gastrointestinal.

[10] Vu D.T., Sethabutr O., Von Seidlein L., Tran V.T., Do G.C., Bui T.C., et al, Detection of Shigella by a PCR assay targeting the ipaH gene suggests increased prevalence of shigellosis in Nha Trang, Vietnam. J Clin Microbiol, 2004 42(5): p. 2031–5. 10.1128/JCM.42.5.2031-2035.2004 15131166 PMC404673

[11] Malorny B., Paccassoni E., Fach P., Bunge C., Martin A., and Helmuth R., Diagnostic real-time PCR for detection of Salmonella in food. Appl Environ Microbiol, 2004 70(12): p. 7046–52. 10.1128/AEM.70.12.7046-7052.2004 15574899 PMC535175

[12] de Boer R.F., Ott A., Guren P., van Zanten E., van Belkum A., and Kooistra-Smid A.M., Detection of Campylobacter species and Arcobacter butzleri in stool samples by use of real-time multiplex PCR. J Clin Microbiol, 2013 51(1): p. 253–9. 10.1128/JCM.01716-12 23152553 PMC3536235

[13] van Maarseveen N.M., Wessels E., de Brouwer C.S., Vossen A.C., and Claas E.C., Diagnosis of viral gastroenteritis by simultaneous detection of Adenovirus group F, Astrovirus, Rotavirus group A, Norovirus genogroups I and II, and Sapovirus in two internally controlled multiplex real-time PCR assays. J Clin Virol, 2010 49(3): p. 205–10. 10.1016/j.jcv.2010.07.019 20829103

[14] Pavlovic M., Huber I., Skala H., Konrad R., Schmidt H., Sing A., et al, Development of a multiplex real-time polymerase chain reaction for simultaneous detection of enterohemorrhagic Escherichia coli and enteropathogenic Escherichia coli strains. Foodborne Pathog Dis, 2010 7(7): p. 801–8. 10.1089/fpd.2009.0457 20156086

[15] Verweij J.J., Laeijendecker D., Brienen E.A., van Lieshout L., and Polderman A.M., Detection of Cyclospora cayetanensis in travellers returning from the tropics and subtropics using microscopy and real-time PCR. Int J Med Microbiol, 2003 293(2–3): p. 199–202. 10.1078/1438-4221-00252 12868656

[16] Verweij J.J., Blange R.A., Templeton K., Schinkel J., Brienen E.A., van Rooyen M.A., et al, Simultaneous detection of Entamoeba histolytica, Giardia lamblia, and Cryptosporidium parvum in fecal samples by using multiplex real-time PCR. J Clin Microbiol, 2004 42(3): p. 1220–3. 10.1128/JCM.42.3.1220-1223.2004 15004079 PMC356880

[17] Lambertz S.T., Nilsson C., Hallanvuo S., and Lindblad M., Real-time PCR method for detection of pathogenic Yersinia enterocolitica in food. Appl Environ Microbiol, 2008 74(19): p. 6060–7. 10.1128/AEM.00405-08 18708521 PMC2565965

[18] Jensen A.N., Andersen M.T., Dalsgaard A., Baggesen D.L., and Nielsen E.M., Development of real-time PCR and hybridization methods for detection and identification of thermophilic Campylobacter spp. in pig faecal samples. J Appl Microbiol, 2005 99(2): p. 292–300. 10.1111/j.1365-2672.2005.02616.x 16033460

[19] Murphy C.N., Fowler R.C., Iwen P.C., and Fey P.D., Evaluation of the BioFire FilmArray(R) GastrointestinalPanel in a Midwestern Academic Hospital. Eur J Clin Microbiol Infect Dis, 2017 36(4): p. 747–754. 10.1007/s10096-016-2858-7 27957599

[20] Cybulski R.J. Jr., Bateman A.C., Bourassa L., Bryan A., Beail B., Matsumoto J., et al, Clinical impact of a Multiplex Gastrointestinal PCR Panel in Patients with Acute Gastroenteritis. Clin Infect Dis, 2018. 10.1093/cid/ciy357 29697761

[21] Rand K.H., Tremblay E.E., Hoidal M., Fisher L.B., Grau K.R., and Karst S.M., Multiplex gastrointestinal pathogen panels: implications for infection control. Diagn Microbiol Infect Dis, 2015 82(2): p. 154–7. 10.1016/j.diagmicrobio.2015.01.007 25796558

[22] Crobach M.J., Planche T., Eckert C., Barbut F., Terveer E.M., Dekkers O.M., et al, European Society of Clinical Microbiology and Infectious Diseases: update of the diagnostic guidance document for Clostridium difficile infection. Clin Microbiol Infect, 2016 22 Suppl 4: p. S63–81. 27460910 10.1016/j.cmi.2016.03.010

[23] McDonald L.C., Gerding D.N., Johnson S., Bakken J.S., Carroll K.C., Coffin S.E., et al, Clinical Practice Guidelines for Clostridium difficile Infection in Adults and Children: 2017 Update by the Infectious Diseases Society of America (IDSA) and Society for Healthcare Epidemiology of America (SHEA). Clinical infectious diseases: an official publication of the Infectious Diseases Society of America, 2018 66(7): p. 987–994. 29562266 10.1093/cid/ciy149

[24] Wong Y.K.N., Gonzalez-Orta M., Saldana C., Cadnum J.L., Jencson A.L., and Donskey C.J., Frequency of Positive Enzyme Immunoassay for Toxin in Stool of Asymptomatic Carriers of Clostridium difficile. Clinical infectious diseases: an official publication of the Infectious Diseases Society of America, 2019 68(4): p. 711–711. 30137260 10.1093/cid/ciy701

[25] Schreckenberger P.C. and McAdam A.J., Point-Counterpoint: Large Multiplex PCR Panels Should Be First-Line Tests for Detection of Respiratory and Intestinal Pathogens. J Clin Microbiol, 2015 53(10): p. 3110–5. 10.1128/JCM.00382-15 25762770 PMC4572537

[26] Freeman K., Mistry H., Tsertsvadze A., Royle P., McCarthy N., Taylor-Phillips S., et al, Multiplex tests to identify gastrointestinal bacteria, viruses and parasites in people with suspected infectious gastroenteritis: a systematic review and economic analysis. Health Technol Assess, 2017 21(23): p. 1–188. 10.3310/hta21230 28619124 PMC5494512

[27] *PHE* *UK Standards for Microbiology Investigations: Gastroenteritis and Diarrhoea: SMI S7 Issue 1* 2013 [cited 2018 September 17th]; Available from: www.gov.uk/government/uploads/system/uploads/attachment_data/file/344110/S_7i1.pdf.

[28] Barratt J.L., Harkness J., Marriott D., Ellis J.T., and Stark D., A review of Dientamoeba fragilis carriage in humans: several reasons why this organism should be considered in the diagnosis of gastrointestinal illness. Gut Microbes, 2011 2(1): p. 3–12. 10.4161/gmic.2.1.14755 21637013

[29] Shea S., Kubota K.A., Maguire H., Gladbach S., Woron A., Atkinson-Dunn R., et al, Clinical Microbiology Laboratories' Adoption of Culture-Independent Diagnostic Tests Is a Threat to Foodborne-Disease Surveillance in the United States. J Clin Microbiol, 2017 55(1): p. 10–19. 10.1128/JCM.01624-16 27795338 PMC5228220

[30] Buss S.N., Leber A., Chapin K., Fey P.D., Bankowski M.J., Jones M.K., et al, Multicenter evaluation of the BioFire FilmArray gastrointestinal panel for etiologic diagnosis of infectious gastroenteritis. J Clin Microbiol, 2015 53(3): p. 915–25. 10.1128/JCM.02674-14 25588652 PMC4390666

