# Supplementary figures and images for: Impact of the BioFire FilmArray gastrointestinal panel on patient care and infection control
Source: PLoS One. 2020 Feb 6;15(2):e0228596. doi: 10.1371/journal.pone.0228596 (PMC7004333; doi:10.1371/journal.pone.0228596)

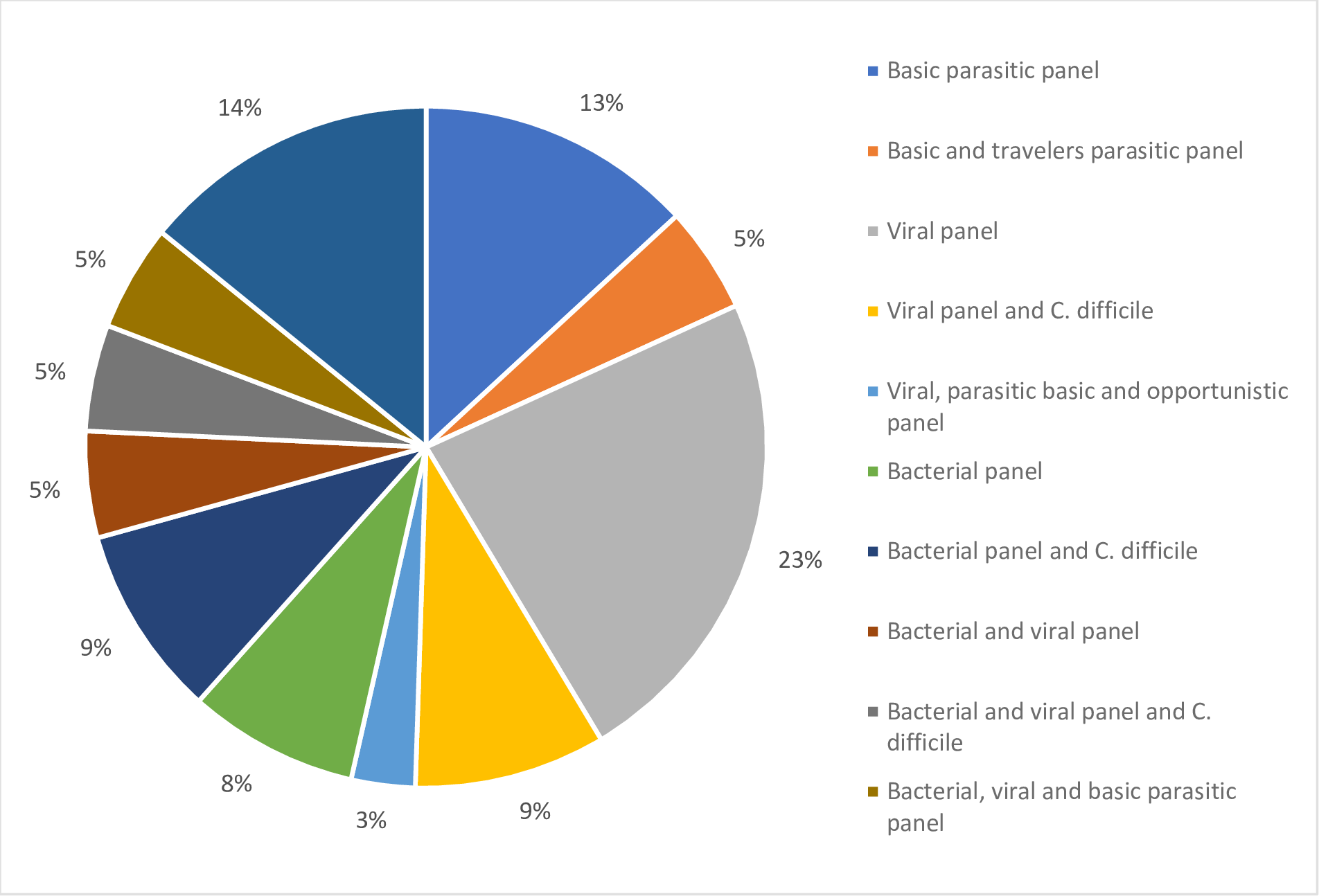

Supplement: S1 Fig — Overview of the requested panels by the clinician, the combinations and the percentages. (TIF) [file pone.0228596.s001.tif]

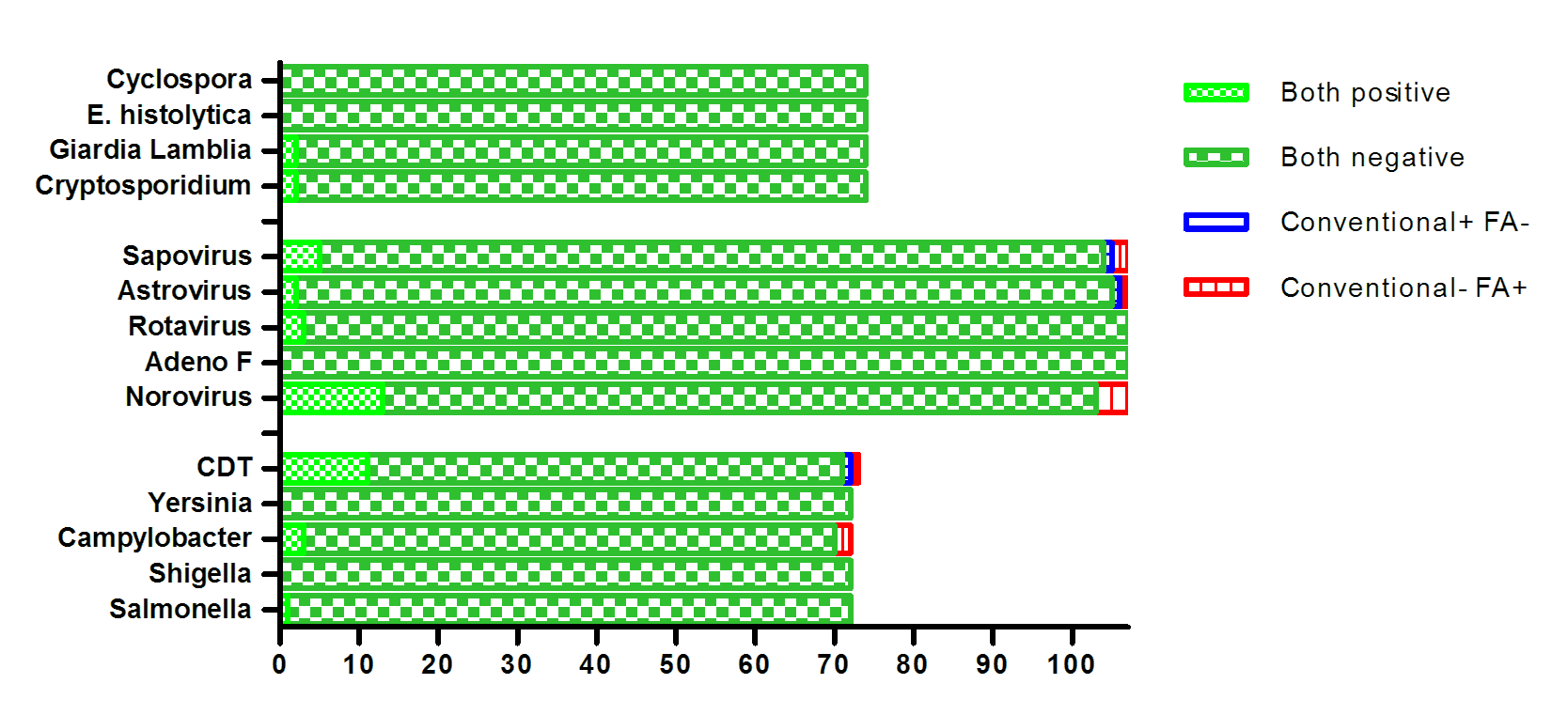

Supplement: S2 Fig — Overview of the concordance between the routine PCR and the FilmArray for the pathogens that are included in both PCR systems. (TIF) [file pone.0228596.s002.tif]
