# Supplementary material for: Impact of the BioFire FilmArray gastrointestinal panel on patient care and infection control
Source: PLoS One. 2020 Feb 6;15(2):e0228596. doi: 10.1371/journal.pone.0228596 (PMC7004333; doi:10.1371/journal.pone.0228596)
Supplement: S1 File — (PDF) [file pone.0228596.s003.pdf]

Impact of the BioFire FilmArray Gastrointestinal Panel on patient care and infection control

J. D. Machiels[1], A. J. H. Cremers[1], M. C. G. T. van Bergen-Verkuyten[1], S. J. M. Paardekoper-Strijbosch[1], K. C. J. Frijns[1], H. F. L. Wertheim[1], J. Rahamat-Langendoen[1], W. J. G. Melchers[1]

[1]Department of Medical Microbiology & Radboudumc Center for Infectious Diseases, Radboud university medical center, Nijmegen, The Netherlands

Introduction

- Admitted patients with suspected gastroenteritis are isolated until the result of the diagnostic test is known.
- The diagnosis of infectious gastroenteritis is generally driven by pathogen directed molecular panels.
- These panels are requested by the clinician based on symptoms, history taking and clinical assessment.
- The pattern of symptoms however cannot differentiate between the causes of gastroenteritis, leading to misdiagnosed cases.
- The BioFire FilmArray GI Panel, a random access test, contains 22 pathogens in a single panel and has a turnaround time of about one hour.

Objective
To evaluate the potential impact of rapid microbial diagnosis in gastrointestinal disease on infection control and patient care.

Methods

- Stool samples of patients with gastroenteritis were tested using routine PCR and the BioFire FilmArray.
- Patient data regarding infection control and patient management were collected.
- The potential impact of implementation the FilmArray in routine diagnostics was assessed.

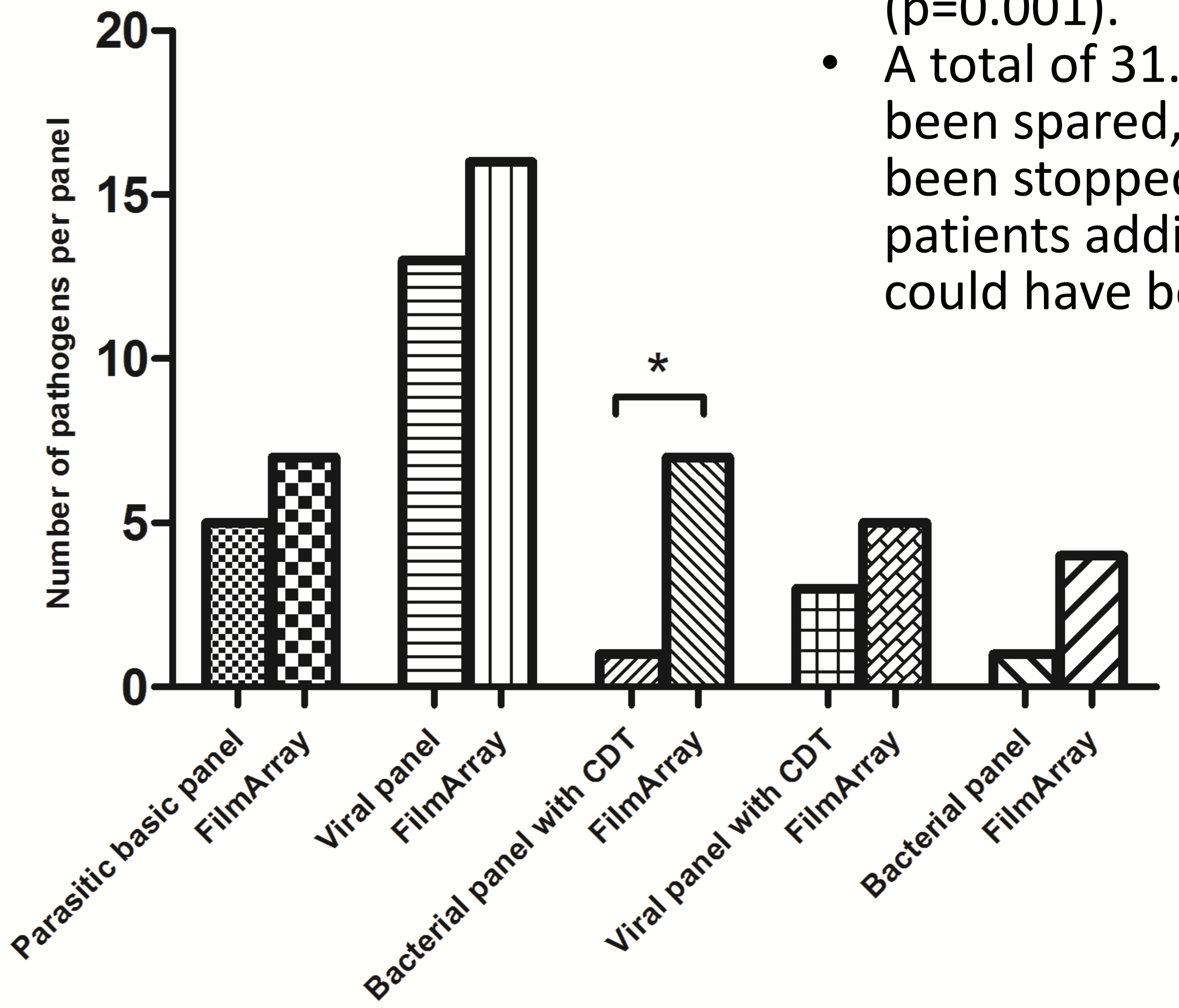

Pathogens found in the routine PCR compared to the BioFire
* Significant result p<0.05

Results

- 182 patients were included. 120 (65,9%) patients were hospitalized. Of the hospitalized patients, 98 (82.4%) were put in isolation.
- Routine PCR detected at least one pathogen in 52 (28,6%) patients, compared to 72 (39,6%) pathogens detected by the FilmArray (p=0.001).
- A total of 31.4 days in isolation could have been spared, antibiotic therapy could have been stopped in three patients and in five patients additional diagnostic procedures could have been prevented.

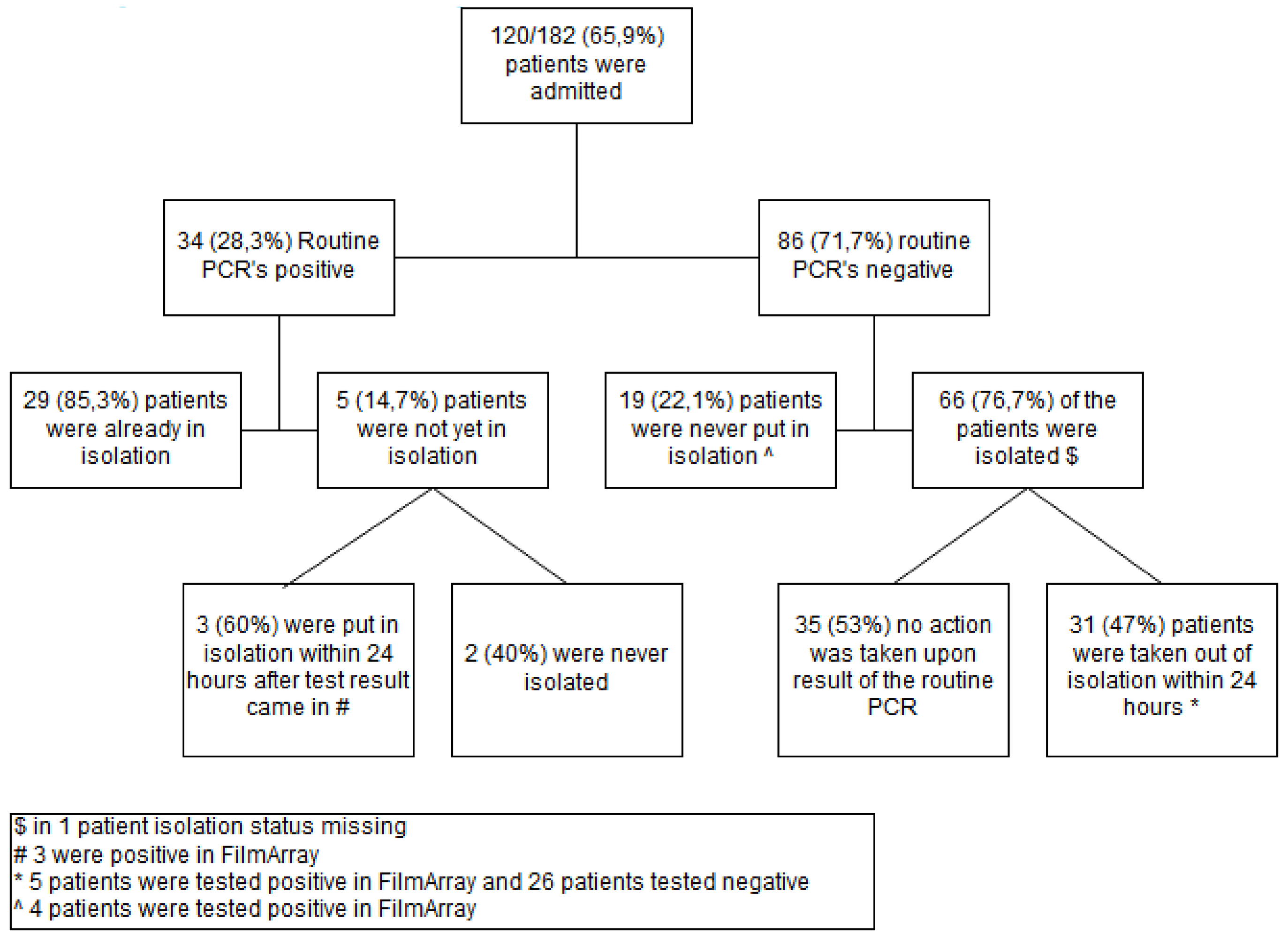

Flow chart of the admitted patients and isolation status

Conclusion
The implementation of the BioFire FilmArray GI panel has significant impact on patient management, reducing unnecessary infection control measures and guiding patient care.

J. D. Machiels
Julian.machiels@radboudumc.nl

Center for Infectious Diseases
Radboudumc
